# Motor-vehicle Crash Patient Injury Patterns from a Level One Trauma Center in a Metropolitan City: A Cross-Sectional Study

**DOI:** 10.7759/cureus.4073

**Published:** 2019-02-14

**Authors:** Obada Hasan, Sadaf Sheikh, Anam Fatima, Asad Abbas, Nida Zahid, Naveed Baloch

**Affiliations:** 1 Orthopaedics, Aga Khan University Hospital, Karachi, PAK; 2 Emergency Medicine, Aga Khan University Hospital, Karachi, PAK; 3 Neurosurgery, Lahore General Hospital, Lahore, PAK; 4 Epidemiology and Public Health, Aga Khan University Hospital, Karachi, PAK; 5 Surgery, Aga Khan University Hospital, Karachi, PAK

**Keywords:** motorcycle accidents, injury pattern, orthopedic, emergency medicine

## Abstract

Introduction

Motorcycles are an inexpensive and popular mode of transportation in Karachi, Pakistan, despite the increasing number of motor vehicle injuries. Although motorcycle-related injuries have been studied previously, to our knowledge, there are no published reports on the orthopedic injuries associated with motorcycles crashes.

Methods

We conducted a cross-sectional study of medical records of patients with motorcycle-related injuries in 2015, at the Aga Khan University in Karachi, Pakistan. We reviewed the patient demographic and medical data, helmet use, the Glasgow coma score, the spectrum of injuries, length of stay, specific injury diagnosis, and final disposition of patients. Data were analyzed using IBM SPSS Statistics for Windows, Version 20.0 (IBM Corp., Armonk, NY).

Results

We identified 450 motorcycle crash injuries. Ninety percent of these victims were males, and 81% were driving at the time of the crash. More than 50% of crashes involved patients age under 29 years, and most crashes (35%) involved patients in the third decade of life. We observed that 6.8% of accidents involved patients younger than 10 years of age. For all the motorcycle-related injuries, 45% occurred during the weekend (Saturday and Sunday). Helmets were worn by only 7% of patients. The most common types of collisions were motorcycle versus car followed by lone motorcycle fall. Of the injuries sustained, the cumulative frequency of orthopedic injuries was the highest (63%), of which 34% were lower limb injuries and 29% were upper limb injuries. The second highest frequency of injuries were head injuries (46%). We also found that 85% motorcycle injury victims sustained fractures.

Conclusion

The frequency of motorcycle-related injuries was high among young adults, and closed fractures of the lower limbs were the most common orthopedic injuries related to motorcycle crashes. Use of helmets among motorcyclists needs to be reinforced to prevent head injuries. We suggest motorcyclists to use protective clothes and motorcycle sidebars to prevent fractures of the lower limb. Further research is needed to determine the type of fractures, type of head injuries, surgical interventions required, and morbidity and mortality in motorcycle-related injuries and whether designing separate lanes for motorcycles will reduce the burden of these injuries on the healthcare system.

## Introduction

Injuries associated with motorcycle crashes are of growing public health importance worldwide. The use of motorcycles has grown more rapidly in Asia. Pakistan, for instance, has had a phenomenal jump from 100,000 motorcycles in use per year in 2000 to 2 millions motorcycles in use per year, currently [1]. Motorcyclists involved in crashes are seriously injured, given their lack of safety measures such as seat belts, lower limb protectors, and airbags. Hence, these riders are exposed to three times higher risk of injury than the car occupants and 16 times more likely to die in the event of a fall or crash [2]. Our institution is a primary level one trauma facility for a city where motorcycles are a popular means of transportation. The objective of this paper is to review the medical records of patients with motorcycle-related injuries to evaluate the epidemiology and pattern of injuries with a focus on orthopedic injuries.

Moreover, we compared recent local motorcycle injury records with former worldwide data. This study was conducted to gather starting point data that would help the emergency physicians and orthopedic surgeons in anticipation and management of orthopedic injuries associated with motorcycle injuries. Also, this study may be useful for the policymakers who may wish to improve road safety measures in a developing nation.

## Materials and methods

We conducted a descriptive cross-sectional study of medical records of patients with motorcycle-related injuries treated at a tertiary care level one trauma center at a university hospital in 2015. Patients provided informed consent before we included their medical records in our review. We excluded any cases with incomplete medical records or those in which the patient was dead at the time of arrival to the hospital. We required a minimum sample size of 450 patients with motorcycle-related injuries based on the anticipated 50% proportion of injury in this population [3], and we required precision of 5% and a level of significance of 5% to account for an expected 15% non-response rate.

After acquiring the exemption permission from the Ethics Review Committee, patients’ medical records were retrieved from the hospital system. The relevant codes were obtained, and 450 medical records were identified for review. We evaluated the demographic profile, mechanism of the crash, the status of the patient (e.g., rider/pillion rider), helmet use, injury arrival interval, orthopedic injuries, and type of intervention if needed. Other variables included hospital length of stay (LOS) and final disposition of patients.

We analyzed our data using IBM SPSS Statistics for Windows, Version 20.0 (IBM Corp., Armonk, NY). Categorical variables were reported as the frequency with their percentages and assessed using the Chi-squared test of independence or Fisher’s exact test, as appropriate. Quantitative variables were described as mean ± standard deviation (SD) or median (interquartile range [IQR]) after checking for normality assumption and assessed using an independent t-test or Wilcoxon Rank Sum test (Mann-Whitney U test), respectively. P<0.05 was considered statistically significant.

## Results

A total of 355 motorcycle crash cases were included in the study. Table 1 presents patient demographics and motor vehicle injury-related factors. More than 50% of crashes involved patients younger than 29 years among which 35.5% were aged 20 to 29 years. Most of those involved in motorcycle crashes were men (90.4%). More than half of the patients were operating the motorcycle themselves, and 66% were confirmed as not wearing helmets (7% were reported as wearing helmets; helmet use was undetermined in 27% of cases).

**Table 1:** Demographic characteristics and road traffic injury factors (n=355)

Variables	n (%)
Age (years)	
< 10	20 (5.6)
10-19	71 (20.0)
20-29	126 (35.5)
30-39	75 (21.1)
40-49	38 (10.7)
50-59	25 (7.0)
Gender	
Male	321 (90.4)
Female	34 (9.6)
Patient’s status	
Driver	280 (78.9)
Passenger	63 (17.7)
Unknown	12 (3.4)
Helmet Use	
Yes	23 (6.5)
No	218 (61.4)
Unknown	114 (32.1)
Type of collision	
Motorcycle to Motor Vehicle	144 (40.6)
Motorcycle to Motorcycle	34 (9.6)
Motorcycle to Rickshaw	8 (2.3)
Motorcycle to Pedestrian	17 (4.8)
Lone motorcycle (Falling off the bike)	116 (32.7)
Others	36 (10.1)
Arrival to hospital (in hours)	
< 1	38 (10.7)
1-6	185 (52.1)
6-12	37 (10.4)
12-24	17 (4.8)
24-48	22 (6.2)
>48	56 (15.8)
Accident day	
Weekday (Monday-Friday)	194 (54.6)
Weekend (Saturday-Sunday)	161 (45.4)
Accident Time	
06:01 to 12:00	60 (16.9)
12:01 to 18:00	130 (36.6)
18:01 to 00:00	127 (35.8)
00:01 to 06:00	38 (10.7)

Forty-five percent of the motorcycle crashes occurred during the weekend (Saturday and Sunday). More motorcycle injuries occurred from 12 PM to 12 AM, with a cumulative frequency of 72.4%. Approximately half (52%) of the crash victims arrived at the hospital within six hours of the crash. The most common type of crash was a motorcycle versus a car (40.6%); other common types of crash were operator slipping from the motorcycle (32.7%), motorcycle-to-motorcycle collision (10%). The remaining 17% of crashes consisted of motorcycle collisions with pedestrians, donkey carts, rickshaws or walls.

Table 2 shows the types of injuries and surgical interventions among motorcycle crash victims. Most injuries were sustained to the head/skull (66.9%), lower limbs (34.3%), and upper limbs (28.7%). Fractures occurred in 63.6% of motorcycle crashes, and fractures occurred in the following regions in descending order: skull and tibia and fibula (47.5%), radius and ulna (39.2%), clavicle (24.5%), femur (23.8%), maxillofacial fractures (19.4%), foot bones (15.6%), humerus (13.7%), pelvis (13.1%), hand bones (14.7%), scapula (7.8%) and spine/vertebrae (4.2%).

**Table 2:** Types of injuries and surgical interventions among motorcycle crash victims (n=355)

Types of injuries	n (%)
Head/Skull	235 (66.19)
Maxillofacial	69 (19.4)
Chest	25 (7.0)
Abdomen and internal viscera	24 (6.8%)
Spine	15 (4.2)
Upper limb	102 (28.7%)
Clavicle	25 (24.5)
Scapula	8 (7.8)
Humerus	14 (13.7)
Radius	25 (24.5)
Ulna	15 (14.7)
Hand	15 (14.7)
Lower limb	122 (34.3)
Pelvis	16 (13.1)
Femur	29 (23.8%)
Tibia	41 (33.6)
Fibula	17 (13.9)
Foot	19 (15.6)
Joint dislocation	4 (1.1)
Fracture type	
Open	53 (14.9)
Closed	173 (48.7)
Not applicable	129 (36.3)
Surgical intervention	261 (73.5)
Craniotomy	21 (7.8)
Debridement	60 (22.3)
Primary fixation	96 (35.7)
Staged fixation	21 (7.8)
Amputation	8 (3.0)
Other	63 (23.4)
Hospital stay (days)	
1-5	249 (70.1)
6-10	59 (16.6)
11-15	22 (6.2)
16-20	10 (2.8)
21-30	7 (2.0)
31-40	3 (0.8)
Not applicable	5 (1.4)
Disposition	
Admitted for observation	147 (41.4)
Admitted and operated	190 (53.5)
Died in the emergency department	1 (0.3)
Died in the ward or intensive care unit	10 (2.8)
Left against medical advice	6 (1.7)
Unknown	1 (0.3)

Analyzing the open fractures of the lower limbs, we noted that open tibia and fibula fractures were the most common (63.6%). About 74% of victims required surgical intervention, most of which were primary fixations (35.7%), debridement (22.3%), craniotomies (7.8%), stage fixation (7.8%), and amputation (3%). The remaining 23.4% of cases required minor procedures like chest thoracostomy, skin or skeletal traction, and laceration repair.

Most patients (70%) had a hospital LOS of one to five days. The final disposition of patients was as follows: admitted for observation only (41.4%), admitted and operated (53.5%), died in the emergency room (0.3%), died in the ward or intensive care unit (3%), or left against medical advice (1.7%).

## Discussion

Many studies have considered the injury spectrum in motorcycle crashes, but there is a paucity of data regarding orthopedic motorcycle injuries in the last five years. Motorcycle use is a primary means of transportation in developing countries such as ours.

According to the first road traffic injuries surveillance report in 2011, the annual incidence of road traffic injuries was 184.3/100,000 population, and death was 5.7/100,000 population [1]. This highlights the crucial requirement to undertake effective local policies in the hope of decreasing motorcyclist injuries. Our data suggested a higher rate of head injuries, maxillofacial injuries, and long bone fractures than those reported in previous epidemiological studies [1]. Furthermore, head and maxillofacial injury incidences were higher than those found in Western countries [2].

Our findings of a higher proportion of men in their young, productive age being victims in motorcycle crashes align with the study by Monk et al., who reported that motor vehicle crashes in victims in their third decade of life resulted in higher fatality rates than those in other decades [4].

Talving et al., in their Los Angeles Countywide trauma registry study of two years, evaluated the association between age, incident timing, and crash severity [5]. He concluded that elder victims sustained more significant trauma to the head, vertebrae, and torso. In contrast, our study showed that younger patients sustained more limb injuries. This is in accordance with Pakistan’s demographic age group [1]. There was a higher risk of serious injury and mortality in this age group that was further potentiated by the lack of use of helmets. This finding aligns with other studies [5]. Reports from developing and developed countries showed lower rates of death and disability in motorcyclists wearing helmets [6-7]; our study had the same conclusions. However, we noticed that many head injuries occurred in those wearing helmets, which calls into question the quality of helmets worn in our population and sold by street vendors at low prices. This corroborates the studies done in Indonesia and California reporting that 45% of riders wore helmets improperly and 48% used nonstandard helmets. This resulted in more victims and higher severity head injuries than crash victims wearing standard helmets or even victims not wearing helmets at all [8-9].

We found a high proportion of injuries in the lower limbs, with tibia and fibula being most common, about half of which were open fractures. This is important for physicians to recognize given that the management of open tibia fractures is difficult and troublesome for both the surgeon and the patient, and these fractures often require multiple surgeries and management of postoperative complications [1].

The most common type of crash in our study was a motorcycle versus a car. Hence, a critical protective measure would be for policymakers to designate motorcycle-only traffic lanes to help separate motorcycle rider/operators from car traffic. Use of motorcycle-only lanes significantly reduces accidents for motorcyclists by as much as 39%, which indicates motorcycle lanes can be a highly successful public safety measure [10].

Our study was limited in its retrospective, single-center design. While additional studies using multiple centers, larger populations, and prospective designs may provide further clarity and insights, our study sill offers immediately actionable insights for policymakers to affect change.

## Conclusions

Understanding the injury spectrum and patient age patterns of motorcycle crashes is beneficial in helping emergency physicians, surgeons, and other health care professionals in treatment planning for improved patient outcomes. This information is equally helpful for road traffic administrators in planning effective accident prevention measures and safety campaigns to reduce the overall burden of public injuries due to motorcycle crashes.
